# A Zebrafish Model of Retinitis Pigmentosa Shows Continuous Degeneration and Regeneration of Rod Photoreceptors

**DOI:** 10.3390/cells9102242

**Published:** 2020-10-06

**Authors:** Abirami Santhanam, Eyad Shihabeddin, Joshua A. Atkinson, Duc Nguyen, Ya-Ping Lin, John O’Brien

**Affiliations:** 1 Ruiz Department of Ophthalmology & Visual Science, McGovern Medical School, The University of Texas Health Science Center at Houston, Houston, TX 77030, USA; Eyad.Shihabeddin.1@uth.tmc.edu (E.S.); josh.a.atkinson@gmail.com (J.A.A.); ducmnguyen9@gmail.com (D.N.); Ya-Ping.Lin@uth.tmc.edu (Y.-P.L.); 2 The MD Anderson Cancer Center/UTHealth Graduate School of Biomedical Sciences, Houston, TX 77030, USA

**Keywords:** retinal degeneration, retinal progenitor cell, transgenic, cone, bipolar cell, Müller cell, P23H rhodopsin

## Abstract

More than 1.5 million people suffer from Retinitis Pigmentosa, with many experiencing partial to complete vision loss. Regenerative therapies offer some hope, but their development is challenged by the limited regenerative capacity of mammalian model systems. As a step toward investigating regenerative therapies, we developed a zebrafish model of Retinitis Pigmentosa that displays ongoing regeneration. We used Tol2 transgenesis to express mouse rhodopsin carrying the P23H mutation and an epitope tag in zebrafish rod photoreceptors. Adult and juvenile fish were examined by immunofluorescence, TUNEL and BrdU incorporation assays. P23H transgenic fish expressed the transgene in rods from 3 days post fertilization onward. Rods expressing the mutant rhodopsin formed very small or no outer segments and the mutant protein was delocalized over the entire cell. Adult fish displayed thinning of the outer nuclear layer (ONL) and loss of rod outer segments, but retained a single, sparse row of rods. Adult fish displayed ongoing apoptotic cell death in the ONL and an abundance of proliferating cells, predominantly in the ONL. There was a modest remodeling of bipolar and Müller glial cells. This transgenic fish will provide a useful model system to study rod photoreceptor regeneration and integration.

## 1. Introduction

Retinitis Pigmentosa (RP), a genetically-based retinal degenerative disease, causes the death of rod photoreceptors and progressive vision loss, leading to blindness in many patients [1,2]. RP affects about 1 in 4000 people worldwide and results in nearly a billion dollars of added healthcare costs annually to patients in the US alone [3]. RP primarily leads to rod photoreceptor cell death associated with night blindness and peripheral vision loss referred to as “tunnel vision,” followed by cone photoreceptor deterioration and loss of central vision. RP is exceptionally heterogeneous at a genetic level, with more than 66 genes that have been identified to cause RP; this heterogeneity makes RP poorly suitable for gene-targeted therapies [4]. Different modes of inheritance have been reported, including autosomal recessive (arRP), dominant (adRP), and X-linked (xRP) genetic traits [5,6,7]. Among these, rhodopsin mutations account for 30% of adRP cases among Americans of European origin. The P23H (Proline to Histidine) opsin mutation is the most common cause of adRP, accounting for ~10% of adRP cases in the USA [4,8,9]. The P23H mutation leads to accumulation of misfolded rhodopsin in the endoplasmic reticulum, activating the unfolded protein response and leading to its proteasomal and lysosomal degradation [10,11]. This leads to the loss of photoreceptors and eventually results in blindness.

Several transgenic rodent models of RP have been established to study the molecular mechanisms involved in the disease progression that leads to the death of photoreceptors [12,13,14,15,16,17]. With some variations between models, these animals share common features of progressive rod photoreceptor degeneration and visual deficits, most with eventual loss of cones as well. A P23H rhodopsin knock-in mouse model more closely mimicked the human retinopathy, with relatively slow degeneration and relative sparing of cone function at early ages [18], providing an excellent model system to study the mechanisms of degeneration. 

A common feature of mammalian model systems is their failure to produce an effective regenerative response to the presence of disease or injury [19,20]. In these models, at the detection of insult to retinal neurons, Müller glial cells (MGCs) exhibit signs of reactive gliosis. While this has an important neuroprotective function in the retina, reactive gliosis inhibits retinal regeneration [21,22]. Unlike mammals, teleost fish have a remarkable capacity to regenerate damaged retina following a variety of insults that destroy neurons. In the teleost retina, MGCs will detect insults to neurons and divide asymmetrically to produce multipotent progenitor cells, which then migrate and differentiate to replace the lost neurons [20,22,23]. 

Zebrafish is a very well-established vertebrate model system with several advantages including rapid development, easy gene manipulation, and high fecundity [24]. Human retinas contain more rods than cones (∼95% rods and ∼5% cones), which is similar to mice; however, the fovea, the central area of the retina responsible for visual acuity, is primarily populated by cones [25]. Zebrafish have a cone-dominated retina (∼65% rods and ∼35% cones) [26], similar to the central human retina. Furthermore, zebrafish retina displays regional cellular and molecular specializations of cones that are similar to those of primate fovea [27]. Therefore, the zebrafish retina provides a useful model system to investigate the molecular mechanisms involved in the development and regeneration of the central retina, which is most relevant for human vision. Zebrafish retinal degenerative models provide research studies with opportunities not only to characterize the disease but also to identify mechanisms of photoreceptor regeneration. 

In the current study we have generated and characterized a P23H rhodopsin transgenic zebrafish line that recapitulates the clinical model of P23H adRP. The transgenic fish show the expression of mutant rhodopsin from 3 days post-fertilization through the adult stages of development, providing a model of chronic rod degeneration similar to RP. We show that rod photoreceptors are continuously being degenerated and regenerated. There are also some qualitative changes in the cone photoreceptors. Overall, the current model provides a unique tool to understand the molecular cues driving the regeneration of photoreceptors in a chronic condition like RP.

## 2. Materials and Methods

### 2.1. Animal Husbandry

Rearing, breeding, and staging of zebrafish (*Danio rerio*) were performed according to standard methods [28]. Wild type AB zebrafish were purchased from the Zebrafish International Resource Center (ZIRC; Eugene, OR, USA), raised, bred, and maintained on a 14 h light/10 h dark cycle. Randomly selected adult and juvenile fish of both sexes were used for experiments. All procedures employing animals have been reviewed and approved by the Institutional Animal Care and Use Committee at the University of Texas Health Science Center at Houston under protocols HSC-AWC-15-0057 and HSC-AWC-18-0047. Transgenic animals developed in this study are available from the investigators.

### 2.2. Transgene Construction and Development of Transgenic Fish

**Figure 1:**
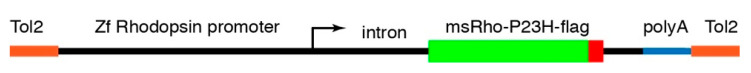
P23H mutant rhodopsin transgene construct. Mouse rhodopsin carrying the P23H mutation and a C-terminal Flag tag is driven by a 1.8 kb Zebrafish rhodopsin promoter.

A myc-DDK-tagged mouse Rhodopsin cDNA clone in pCMV6 vector was purchased from OriGene (Rockville, MD, USA). The P23H mutation was inserted in this clone using a QuickChange site-directed mutagenesis kit (Agilent, La Jolla, CA, USA) and primers MsOpsP23H F—TGGTGCGGAGCCACTTCGAGCAGCC and MsOpsP23H R—GGCTGCTCGAAGTGGCTCCGCACCA according to the manufacturer’s protocol. The Tol2 transgene plasmid pT2AL200R150G [29] and Tol2 transposase cDNA plasmid pCS-TP [30] were generously provided by Dr. Koichi Kawakami (National Institute of Genetics, Mishima, Japan). A 1.8 kb Zebrafish rhodopsin promoter clone was generously provided by Dr. Xinping C. Zhao (University of Texas Health Science Center at Houston). The Zebrafish rhodopsin promoter was amplified with primers JOB316—TCACTTGGGCCCGGCTCGAGCATGTCAGAAGC and JOB317—CTCAGGATCGGTCGACCTGCAGGGCGCTCAGCCCCTTCTGC using Phusion DNA polymerase (New England Biolabs, Natick, MA, USA) and cloned into XhoI and PstI sites of pT2AL200R150G using Cold Fusion cloning (System Biosciences, Palo Alto, CA, USA). Clones were sequenced on both strands to confirm the insert. The mouse Rhodopsin cDNA harboring the P23H mutation was amplified by PCR with primers JOB318—CAAAGAATTCCTCGACGGATCCGGTACCGAGGAGATCTG and JOB319—CATGTCTGGATCATCATCGATCCCGGGATCTGTTCAGGAAACAG using Phusion DNA polymerase and cloned into the Tol2 zebrafish rhodopsin promoter construct at BamHI and ClaI restriction sites using Cold Fusion cloning. The final constructs (pT2-Dre-rho:Mmu-Rho(P23H)Flag) were sequenced on both strands. The transgene construct is illustrated diagrammatically in Figure 1. 

To generate transgenic fish, Tol2 transposase mRNA made by in vitro transcription of the pCS-TP plasmid using the mMessage mMachine kit (Life Technologies, Austin, TX, USA) and the finished transgene DNA were co-injected into 1 cell stage AB strain zebrafish embryos. The resulting fish were grown to adulthood, outcrossed to wild-type AB zebrafish and pools of embryos screened for transgene transmission by PCR using primers JA1—GCAGCTGGTCTTCACAGTCAAG and JA2—TTGTAATCCAGGATATCATTTGCTG or JA3—CACTCAAGCCTGAGGTCAACAAC and JA4—GAGTTTCTGCTCGAGCGGC. Both sets of primers span the region from mouse rhodopsin to the tag sequences and are specific for the transgene. Fish that transmitted the transgene to offspring were bred further to establish stable transgenic lines. 

One line that displays good transgene expression has been propagated by incrossing and used for the studies reported here. Transgenic fish were genotyped by PCR of tail cut DNA as described by Meeker et al. [31]. The clipped tail piece was digested in 100 µL of 50 mM Sodium hydroxide at 95 °C for twenty minutes and neutralized by addition of 10 µL of 1 M Tris-Cl, pH 8.0. Aliquots of this genomic DNA were amplified by PCR with primers JA1 and JA2 (Figure S1).

### 2.3. BrdU Labeling

Adult Zebrafish of 4–6 months age were anesthetized in 0.02% 3-aminobenzoic acid ethyl ester (Tricaine/MS222; Millipore-Sigma, St. Louis, MO, USA) until unresponsive to touch. Anesthetized fish were injected intraperitoneally with 5-bromo-2-deoxyuridine (BrdU) 5 μL/0.1 g body weight at a concentration of 5 mg/mL BrdU (Sigma B-9285) freshly prepared in sterile PBS. Animals were housed in static small tanks (1.5-L breeding tanks) during treatment and euthanized after 5 or 24 h by immersion in 0.15% Tricaine/MS222 followed by decapitation. A sample size of 3 animals was used for each time point.

### 2.4. Tissue Preparation, Histology, Immunocytochemistry (ICCH) and Imaging

All fish for tissue analysis were collected during the morning, between nine and eleven a.m. Two to nine days post-fertilization (dpf) larvae were anesthetized in 0.15% Tricaine/MS222 and fixed in 4% paraformaldehyde (PFA; Electron Microscopy Sciences, Hatfield, PA, USA) in 0.1 M phosphate buffer, pH 7.4 (PB) for 1 h at room temperature (RT). Afterward, specimens were washed three times at 15 min intervals in PB and infiltrated in 30% sucrose in PB overnight at 4 °C. Larvae were then frozen in Tissue-Tek O.C.T. compound (4583, Sakura Olympus, Italy) using dry ice and stored at −80 °C. Adult zebrafish of age 4–6 months were sacrificed by immersion in 0.15% Tricaine/MS222 followed by decapitation, and their eyes enucleated. Eyes were fixed in either 4% PFA in 0.1 M PB, or ethanolic PFA at a ratio of 9:1 (9 parts 95% ethanol to 1 part 4% PFA) for 1 h at RT. Afterward, specimens were washed four times (15 min intervals) in PB and infiltrated in 30% sucrose in PB overnight at 4 °C. Eyes were then frozen in Tissue-Tek O.C.T. compound using dry ice and stored at −80 °C. Cryostat sections (12 μm thick) were collected on SuperFrost Plus slides (1255015, Fisher Scientific, Waltham, MA, USA) and used for immunocytochemistry (ICCH). ICCH on retinal sections was performed by incubation in (i) blocking solution with 0.3% Triton-X100, 5% of the serum of the species in which the secondary antibody was generated (Donkey Serum or Goat Serum; Jackson ImmunoResearch, West Grove, PA, USA) and 0.01 M Phosphate Buffered Saline (PBS)(P3813, Millipore-Sigma) for 1 h at RT; (ii) primary antibody (Ab) diluted in PBS, 0.1% Triton-X100 and 5% serum overnight at RT; (iii) fluorescent secondary Ab, diluted as the primary Ab, for 1 h at RT. For nuclear counterstaining, retinal sections were mounted in Vectashield with DAPI (H-1000; Vector Laboratories, Burlingame, CA, USA) and coverslipped. The primary and secondary antibodies used in this study are listed in Table 1. Images were taken using a Zeiss LSM 780 laser scanning confocal microscope (Thornwood, NY, USA). 

For wholemount immunostaining experiments with BrdU labeling, the eyes were fixed as described above and an eyecup preparation made as follows. After fixation, a cut was made on the cornea with micro scissors and the cornea and lens removed, leaving an intact retina accessible for antibody penetration. The eyecups were treated in 2N HCl for 30 min at RT before incubation with the primary antibody. The eyecups were incubated with primary antibody for five days at 4 °C in a shaker, followed by four washes with PBS at 15 min intervals. Afterward, the tissue was incubated with a secondary antibody overnight at 4 °C, followed by four washes with PBS at 15 min intervals. The eyecups were transferred to a Petri dish containing 0.5× PBS and processed under a microscope. The retinas were removed and four cuts were made with micro scissors to flatten the retina. The retinas were mounted in Vectashield with DAPI. Images were taken using a Zeiss LSM 780 confocal microscope.

### 2.5. TUNEL Staining 

4% PFA-fixed retinal cryosections of 10–12 µm thickness were washed in PBS for 15 min at RT. Tissues were permeabilized in 100 mM sodium citrate dissolved in PBTx (PBS plus 0.1% Triton X-100) at RT for 2 min, followed by the addition of terminal deoxynucleotidyl transferase-mediated fluorescein-dUTP nick end labeling (TUNEL) mix (in situ cell death detection kit; Roche, Mannheim, Germany) according to the manufacturer’s instructions. After incubation at 37 °C for 1 h inside a humidified chamber, retinal sections were mounted in Vectashield with DAPI and coverslipped. TUNEL-positive cells were visualized by confocal fluorescence microscopy (Zeiss 780). 

### 2.6. Quantitative Real-Time PCR

RNA was isolated from eyecup tissue using Aurum total RNA mini kit (BioRad, Hercules, CA, USA), and the total RNA was extracted according to the manufacturer’s instructions. The cDNA for each retina was synthesized from 75 ng total RNA with the Thermoscript RT–PCR System (BioRad) using oligo (dT) primers according to the manufacturer’s instructions. 

Primers were designed using Primer3 combined with BLAST from NCBI (Bethesda, MD, USA) and primers (Table 2) were synthesized by Integrated DNA Technologies (Coralville, IO, USA). The quantitative measurement of PKC-α (*prkca*) and Glul-a (*glula*) mRNA levels from retina tissue was performed with real-time PCR using a BioRad CFX maestro thermal cycler with the SYBR Green PCR Master Mix (BioRad) in a one-step reaction according to the manufacturer’s instructions. The relative mRNA levels where calculated using the reference housekeeping gene GAPDH expression level. The thermal cycle was programmed for 30 s at 98 °C for initial denaturation, followed by 35 cycles of 10 s at 98 °C for denaturation, 10 s at 59 °C for annealing, 10 s at 72 °C for extension, and 1 min at 72 °C for the final extension. The melting curves and gel electrophoresis of the end products were obtained to confirm the specificities of the PCR reactions. The relative quantification of target genes was determined using the ΔΔCt quantitative RT–PCR method [32]. The primers used are listed in Table 2.

### 2.7. Statistical Analysis

A sample size of six animals was used for nuclei count and outer segment length calculation. An average of measurements from three sections per retina represented one fish. A sample size of three animals per time point was used for counts of proliferating cells labeled with BrdU. A sample size of three animals was used for PKC-α and Glul-a relative mRNA level analysis by quantitative RT-PCR. All samples were prepared in duplicates and the average was used for quantification. All data are represented as the mean ± SD; statistical significance was determined using a two-tailed Student *t*-test from three or more samples. Statistical significance is reported as asterisks in graphs (∗∗∗ for *p* < 0.001, ∗∗ for *p* < 0.01, ∗ for *p* < 0.05).

## 3. Results

### 3.1. Mutant Rhodopsin is Expressed in the Rod Photoreceptors

**Figure 2:**
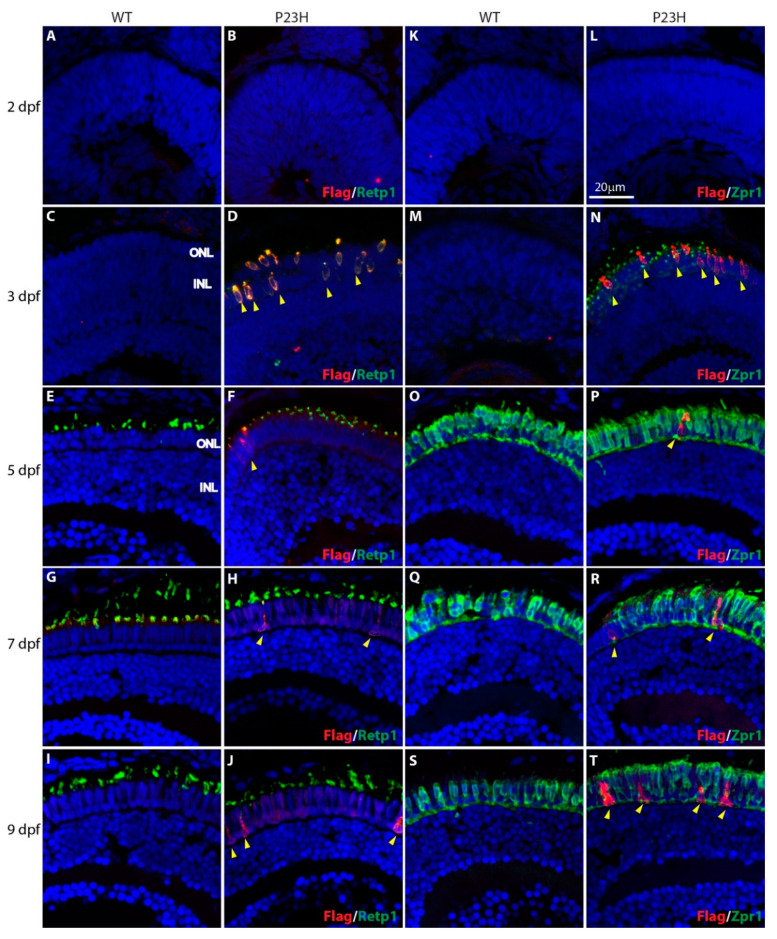
Expression of P23H Flag-tagged rhodopsin at early stages of photoreceptor development in wild type (WT) and P23H transgenic (P23H) zebrafish. Expression of P23H mutant rhodopsin (Flag; red) and rhodopsin (Retp1; green) at 2 dpf (**A**,**B**), 3 dpf (**C**,**D**), 5 dpf (**E**,**F**), 7 dpf (**G**,**H**), and 9 dpf (**I**,**J**). Expression of Flag-tagged P23H mutant rhodopsin (red) compared to double cones (Zpr1, green) at 2 dpf (**K**,**L**), 3 dpf (**M**,**N**), 5 dpf (**O**,**P**), 7 dpf (**Q**,**R**), and 9 dpf (**S**,**T**). Yellow arrowheads denote cells expressing P23H mutant rhodopsin. Nuclei labeled with DAPI are blue. ONL: outer nuclear layer; INL: inner nuclear layer. Scale bar in L applies to all panels.

We developed a Tol2 transgene construct in which a Flag-tagged mouse rhodopsin carrying the P23H mutation is driven by the zebrafish rhodopsin promoter (see Methods and Figure 1). We established stable transgenic lines and have propagated an efficiently-expressing line by incrossing. Immunofluorescence analysis of stable transgenic larvae revealed expression of the P23H mutant rhodopsin, as detected with antibodies against the Flag tag, from the early stages of development (Figure 2). At 2 dpf (Figure 2A,B,K,L) we did not detect expression of rod or cone markers in either wild type (WT) or P23H transgenic zebrafish. At 3 dpf the P23H transgenic fish showed expression of the Flag-labeled mutant rhodopsin, co-labeled with the Retp1 anti-rhodopsin antibody in numerous cells spread throughout the outer nuclear layer (ONL) of the central retina (Figure 2D). These cells did not co-label with the Zpr1 antibody that binds to double cone photoreceptors [33], which instead labeled numerous small structures reminiscent of nascent cone outer segments throughout the central retina (Figure 2N). In contrast, the WT fish at 3 dpf did not show expression of either rod (Retp1) or cone (Zpr1) markers. At 5 dpf (Figure 2F,P), only a few cells expressing the P23H rhodopsin were detected in the ONL near the retinal margin, suggesting that P23H rhodopsin-expressing cells from the initial wave at 3 dpf had been lost. At this age, the cone antigen labeled by the Zpr1 antibody was expressed strongly throughout the ONL, and did not co-localize with the Flag-labeled mutant rhodopsin (Figure 2O,P). At 7 and 9 dpf (Figure 2G–J,Q–T), an increasing number of Flag-labeled cells in the ONL and expanded distribution toward the central retina was evident. Labeling with the Zpr1 antibody showed that the Flag-tagged mutant rhodopsin was not expressed in the double cones at any age (Figure 2M–T).

In 3–9 dpf larvae, the mutant rhodopsin was distributed throughout the labeled cells (Figure 2, yellow arrowheads), revealing a photoreceptor-like morphology including a soma, synaptic terminal and sometimes a very small outer segment. This morphology changed from compact and oval with a centrally-placed nucleus at 3–5 dpf to elongate, with a nucleus placed basally at the border of the outer plexiform layer (OPL) from 7–9 dpf. Double labeling with the Retp1 monoclonal antibody against rhodopsin revealed that the stunted outer segments contained additional rhodopsin (see the yellower color of outer segments, e.g., in Figure 2D,F,H), likely representing the endogenous rhodopsin in the rod outer segments, although the normal elongate outer segment structure did not develop. The delocalized distribution of P23H rhodopsin and stunted outer segments is comparable to that observed in other models of RP [14,15,16,34]. Note that we observed the Retp1 antibody to label outer segments of one of the elements of the double cones, likely representing a cross-reaction with one cone opsin. This accounts for the majority of outer segment labeling in the 5–9 dpf larvae shown in Figure 2E–J. No rods were detected that displayed normal outer segments. An expanded examination of the association of Retp1 labeling with cone outer segments is shown in Figure S2. The Retp1 antibody used in this study recognizes a sequence in the amino terminal 10 amino acids of rat rhodopsin [35]. Sequence alignment showed that rat rhodopsin shared 9 of 10 amino acids with zebrafish rhodopsin and 9 of 10 amino acids with zebrafish green cone opsins (opn1mw1–4), which are localized to double cone outer segments and could lead to cross-reaction.

**Figure 3:**
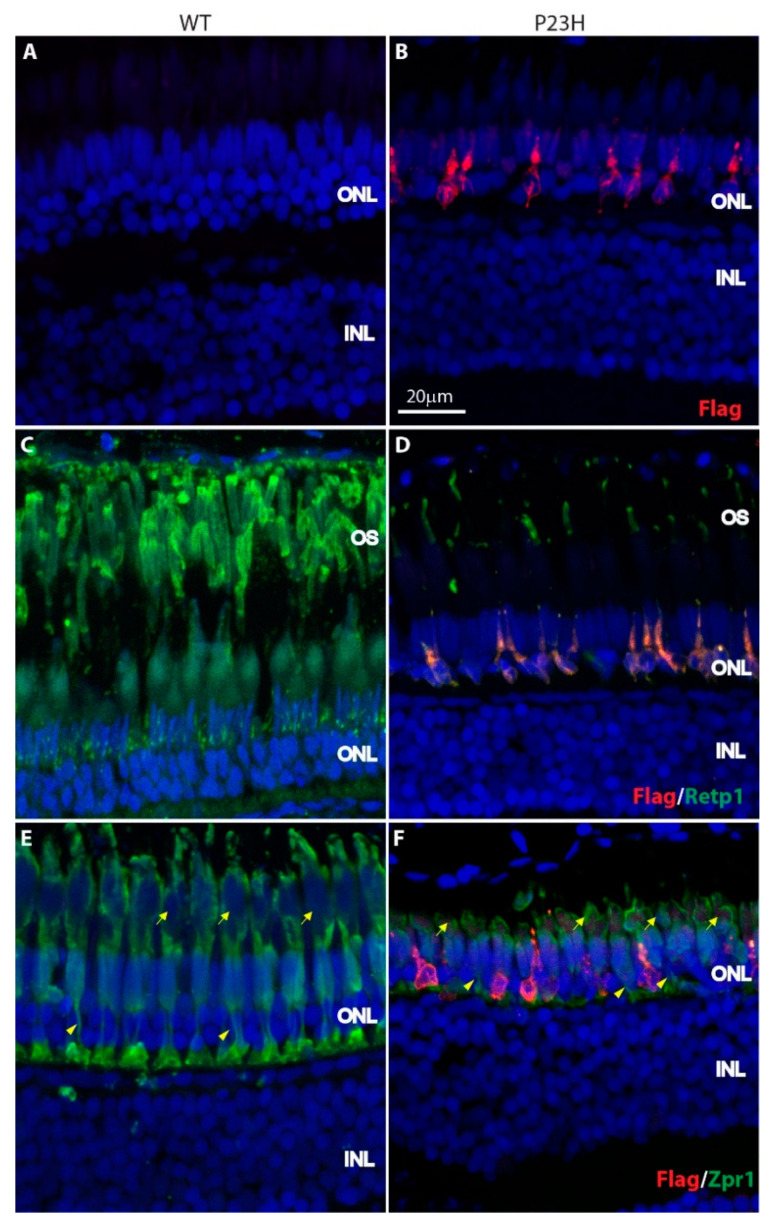
Expression of P23H Flag-tagged rhodopsin in adult zebrafish retina. (**A**,**B**) The Flag-tagged P23H mutant rhodopsin (red) is expressed by sparsely-distributed photoreceptors in the adult fish (**B**); there is no labeling in wild type fish (**A**). (**C**,**D**) Mutant rhodopsin (red) colocalizes with rhodopsin (Retp1, green) in the rods. (**E**,**F**) Mutant rhodopsin (red) is expressed only by the rods and not the cones, as seen with the cone marker Zpr1 (green). Yellow arrowheads represent the cone axons and yellow arrows represent the cone myoids. ONL: outer nuclear layer; INL: inner nuclear layer; OS: outer segments. Scale bar in B applies to all panels.

In adult P23H zebrafish from 4–8 months, expression of mutant rhodopsin was evident in rod-like cells throughout the ONL (Figure 3A,B). Note that the number of cells expressing mutant rhodopsin has increased relative to the few cells in the early development. Labeling with the Retp1 antibody to label total rhodopsin showed that the number and length of outer segments labeled for rhodopsin was greatly reduced in the P23H transgenic fish compared to the wild type fish (Figure 3C,D). Once again, the Retp1 antibody to rhodopsin labeled one of the double cone outer segments (Figure S2), confounding the assessment of whether any rods in the P23H transgenic retina form normal outer segments. However, all rods that expressed the mutant rhodopsin (labeled with anti-Flag antibody) displayed only small, deformed outer segments (Figure 3B,D,F). These were relatively enriched for labeling with Retp1, suggesting that the endogenous wild-type rhodopsin traffics properly to the outer segments.

In order to see whether there was any change in the cone morphology, we labeled with the Zpr1 antibody. The Zpr1 staining revealed that the length of cone axons was shorter in the P23H transgenic animals compared to the WT (Figure 3E,F, arrowheads). The cone myoids and outer segments were also shorter in the P23H compared to the WT (Figure 3E,F, arrows). There was no significant change in the number of double cones labeled with Zpr1 in the P23H transgenic fish with a mean of 34.2 ± 5.6 in the WT vs. 32.4 ± 4.8 in the P23H per ~210 µm image field in the retinal section (*p* = 0.7; n = 3 fish per genotype).

### 3.2. Degeneration of Rod Photoreceptors in the P23H Transgenic Zebrafish

**Figure 4:**
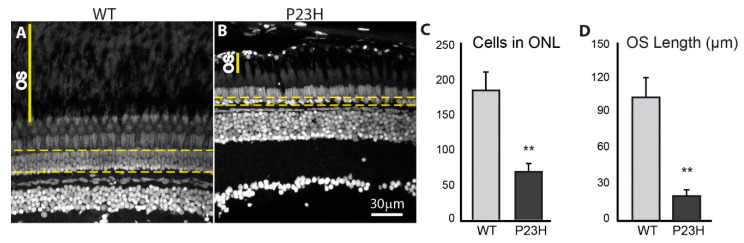
Photoreceptor loss in the P23H transgenic zebrafish. DAPI label of adult retina sections. The yellow dotted lines encompass nuclei in the ONL counted to assess photoreceptor loss. OS indicates the space between photoreceptor myoids and retinal pigmented epithelium. The number of cells in the ONL is almost three times greater in the WT (**A**) than in the P23H mutant (**B**), which usually shows a single irregular layer of nuclei. (**C**) Quantification of photoreceptor counts in WT and P23H transgenic (n = 6 fish per genotype; error bars are ± SD; ** *p* < 0.01). (**D**) Quantification of OS length in WT and P23H transgenic (n = 6 fish per genotype; error bars are ± SD; ** *p* < 0.01). Scale bar in B applies to A and B.

The delocalization of rhodopsin and stunted outer segments observed in the P23H transgenic zebrafish is consistent with characteristics of rod photoreceptors in models of RP that show retinal degeneration [15,16,36]. To assess whether the P23H transgenic zebrafish displayed rod degeneration, we first counted the number of cells in the ONL of adult retina using retinal tissue sections (Figure 4A,B, outlined areas, which exclude elongate double cone and long single cone nuclei, but include short single cones [37]). Figure 4C shows that the number of nuclei in the ONL of the P23H fish was almost three-fold less than the WT. P23H transgenic fish had 67 ± 11 nuclei per ~210 µm image field, while wild type fish had 181 ± 25 nuclei per ~210 µm image field in the retinal section (*p* = 0.005; n = 6 fish per genotype). We usually observe a single irregular layer of nuclei in the ONL of the P23H fish, unlike the regularly arranged, multilayered ONL in WT. Furthermore, the space between photoreceptor myoids and retinal pigmented epithelium, normally occupied by outer segments (OS), was reduced in the P23H to one fifth that of the WT (Figure 4A,B, solid yellow lines). In the WT the OS space was 100 ± 15 µm, whereas in the P23H it was 20 ± 5 µm (*p* = 0.005; n = 6 fish per genotype; Figure 4D). 

**Figure 5:**
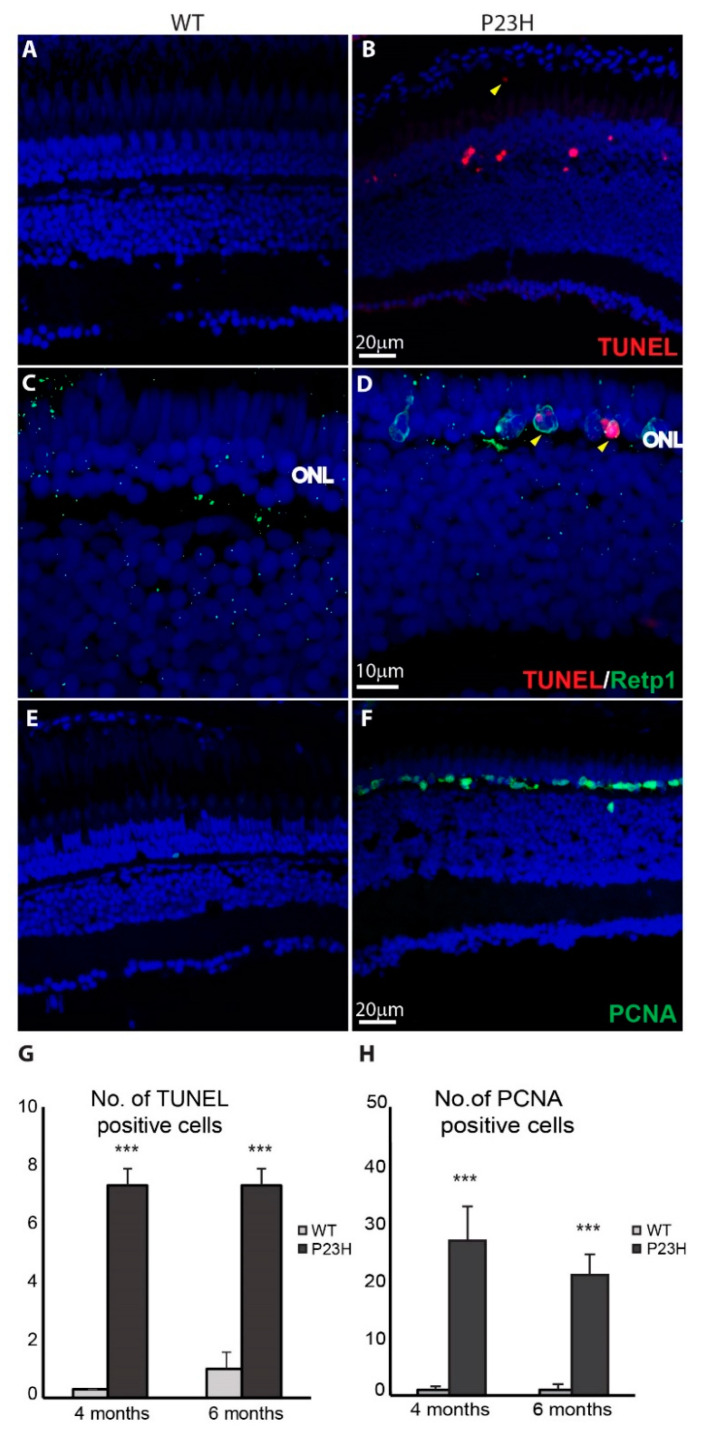
Cell death and cell proliferation in the P23H transgenic zebrafish. (**A**,**B**) Cell death detection using TUNEL staining shows TUNEL-positive dying cells (red) in the P23H zebrafish retina (4 months old). The yellow arrowhead shows a TUNEL-positive cell in the sub-retinal space. (**C**,**D**) Yellow arrowheads show the colocalization of Retp1 (green) with TUNEL labeling in the P23H transgenic fish. (**E**,**F**) PCNA immunostaining shows many PCNA-positive proliferating cells (green) in the ONL of the P23H zebrafish, but very few in WT. ONL: outer nuclear layer. (**G**) Numbers of TUNEL positive cells and (**H**) PCNA-positive cells per 210 µm image field in 4-month old and 6-month old WT and P23H retina (n = 6 fish per genotype; error bars are ± SD; *** *p* < 0.001).

Since we notice loss of cells in the ONL of the P23H transgenic fish, we examined cell death using terminal deoxynucleotidyl transferase dUTP nick end labeling (TUNEL) assays. Figure 5B shows the presence of TUNEL-positive cells (red) in the P23H transgenic, primarily in the ONL, whereas the wild type retina generally did not display any TUNEL-positive cells (Figure 5A). The colocalization of rhodopsin immunolabeling and TUNEL further confirmed that the apoptotic cells in the ONL of the transgenic fish are the rods (Figure 5D). At 4 and 6 months of age, P23H transgenic fish showed a significantly higher number of TUNEL positive cells in the ONL (per ~210 µm image field) compared to the WT (Figure 5G). These results show that apoptosis is one of the forms of cell death happening in the RP model, although we did not exclude other forms of cell death. A few TUNEL-positive cells were also seen in the P23H transgenic in the sub-retinal space (Figure 5B, yellow arrowhead), where phagocytic microglia are seen in mammalian RP models [14,38,39]. We also observed a small number of TUNEL-positive cells in the inner nuclear layer (INL) and in the retinal ganglion cell (RGC) layer of the P23H transgenic retina (Figure S3), suggesting that a few other cells in the retina also undergo cell death in this model.

### 3.3. Regeneration in the P23H Transgenic Zebrafish

Proliferating cell nuclear antigen (PCNA) is essential for replication in eukaryotic cells and is expressed in the nucleus during the DNA synthesis phase of the cell cycle [40]. It is commonly used as a progenitor cell marker. Since teleost fish are capable of regeneration, we assessed the extent of regeneration happening in the P23H transgenic by labeling for PCNA. Figure 5E,F show a large number of PCNA-positive cells decorating the ONL in the P23H fish, compared to very few PCNA-labelled cells in the wild type fish. Unlike acute damage models in the zebrafish retina [41,42], we observed very few PCNA-positive cells in the INL compared to the ONL in the P23H transgenic fish. At 4 and 6 months of age, P23H transgenic fish showed a significantly higher number of PCNA labeled cells in the ONL compared to the WT (Figure 5H). At any given time point we have observed the high expression of PCNA in the P23H transgenic zebrafish during adult ages ranging from 4–12 months (data not shown). The abundance of proliferating cells in the ONL of the P23H transgenic suggests that degenerating rods are being replaced continuously in this model from progenitor cells located in the ONL.

**Figure 6:**
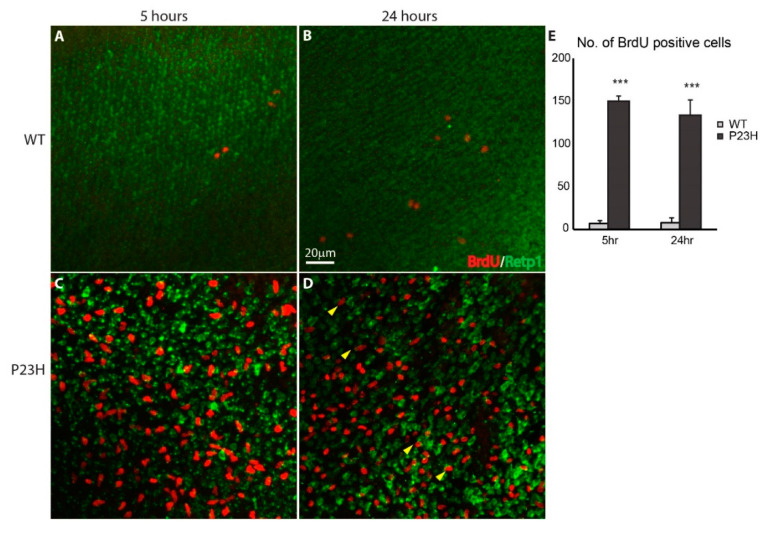
Continuous cell proliferation in the P23H transgenic zebrafish. Wholemount imaging of BrdU (red) labeled cells in ONL of the WT (**A**,**B**) and P23H transgenic (**C**,**D**) 5 and 24 h after BrdU injection respectively. Retp1 (green) labels rhodopsin. Yellow arrowheads in panel D show BrdU-labeled cells that co-label with Retp1, indicating their differentiation into rods. Scale bar in B applies to A–D. (**E**) The number of BrdU-labeled cells in the P23H transgenic fish is significantly higher than in the WT (*n* = 3 fish per genotype; error bars are ± SD; *** *p* < 0.001).

5-bromo-2-deoxyuridine (BrdU) is a nucleoside analog that is specifically incorporated into DNA during S-phase [43] and can subsequently be detected with an anti-BrdU specific antibody. We used a short time BrdU injection to label proliferating cells in order to further assess the regeneration potential of the P23H transgenic line. As seen in the wholemount immunofluorescence imaging in Figure 6, BrdU-positive cells were abundant in the ONL of the P23H transgenic fish 5 and 24 h after BrdU injection (Figure 6C,D), whereas very few cells were labeled in the WT (Figure 6A,B). The large number of BrdU-labeled cells seen just 5 h after BrdU injection demonstrates that proliferation is extensive in the P23H transgenic retina. Furthermore, some BrdU-labeled cells 24 h after injection co-label for rhodopsin (Figure 6D, arrowheads), indicating that proliferating cells are differentiating into rods in this model. Counts of BrdU-labeled cells revealed that the P23H transgenic had significantly more labeled cells than WT: almost 20-fold at 5 h and almost 17-fold at 24 h (n = 3, *p* < 0.001 for both; Figure 6E).

### 3.4. Retina Remodeling in the P23H Transgenic Zebrafish

**Figure 7:**
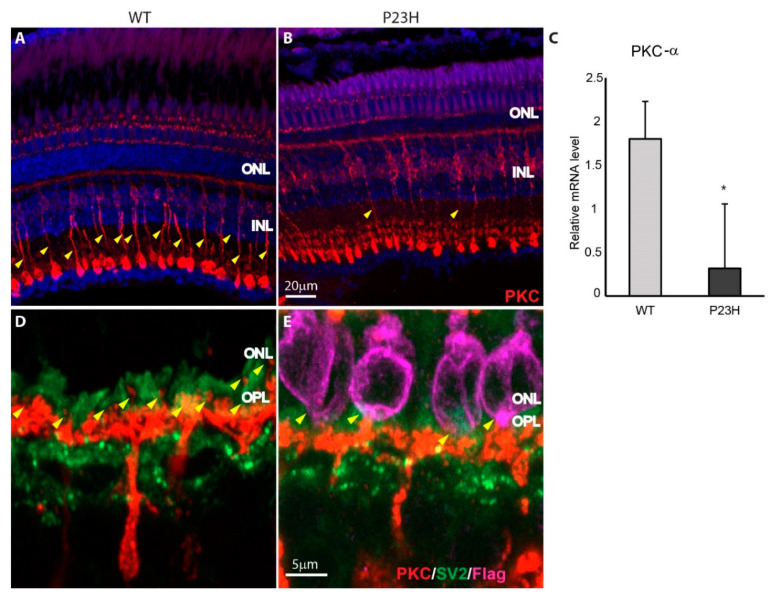
Bipolar cell synapses in P23H transgenic retina. (**A**,**B**) Immunolabeling for PKC-α (red) shows that rod bipolar cells are morphologically similar, but PKC-α labeling is less intense in the P23H transgenic (B) compared to WT (A). Yellow arrowheads indicate the bipolar cell axons. (**C**) Relative mRNA level of PKC-α is higher in the WT compared to the P23H (*n* = 3 fish per genotype; error bars are ± SD; * *p* < 0.05). (**D**,**E**) Enlarged images show the synaptic contacts made by PKC-labeled bipolar cells with rod and cone photoreceptor terminals labeled for SV2 (green). Fine synaptic contacts onto rods are shown with yellow arrowheads. Bipolar cells appear to contact some of the rods expressing the P23H rhodopsin (magenta) (**E**). ONL: outer nuclear layer; INL: inner nuclear layer; OPL: outer plexiform layer.

Since the rod cells are dying and new cells are produced, we were interested to see the rod bipolar cell connections and the synapses they form. The anti-PKC-α antibody was used to label the rod bipolar cells and anti-SV2 antibody to label all photoreceptor terminals. Note that the anti-PKC-α antibody is not specific for one single zebrafish conventional PKC, but rather detects a combination of PKC-α and -β variants [44]. PKC staining was generally weaker in the P23H than in the WT (Figure 7A,B), largely as a result of weaker expression in the Mb1 rod bipolar cells. This was particularly apparent in the weaker labeling of the descending axons of the Mb1 bipolar cells (Figure 7A,B arrowheads) that connect the large, round axon terminals at the bottom of the inner plexiform layer (IPL). An orthogonal projection further confirmed that the PKC immunostaining was less intense in the P23H compared to the WT, especially in the IPL region (Figure S4). There was a similar number of Mb1 bipolar cells in the P23H and WT retina, as assessed by the number of large terminals present at the bottom of the IPL (WT: 15.4 ± 2.3 terminals per 210 µm image field, *n* = 3; P23H: 14.8 ± 1.0 terminals per field, *n* = 3; *t*-test, *p* = 0.68). However, the PKC-α mRNA levels from the whole retina tissue showed a significant decrease in P23H compared to WT (*n* = 3; *p* = 0.03; Figure 7C).

A closer look at the photoreceptor synaptic junction (Figure 7D,E) shows that in WT there are many fine bipolar cell dendrites extending past cone terminals (synaptic vesicle protein SV2—green) to make small synaptic contacts in the OPL and proximal ONL with rod terminals weakly labeled for SV2 (Figure 7D arrowheads). These fine dendrites were not seen in the P23H transgenic (Figure 7E), although short bipolar cell processes appeared to make contacts with some of the rods. This change in the immunostaining of PKC and synaptic vesicles in the P23H transgenic is probably due to the reduced number of rod terminals and the continuous remodeling of the ONL by dying and newly regenerated rod photoreceptors.

**Figure 8:**
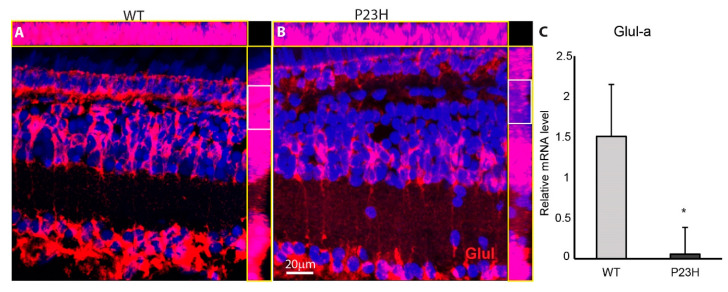
Glutamine synthetase (Glul) immunostaining is weak in the P23H transgenic fish. (**A**,**B**) Immunolabeling for Glul (red) shows the overall intensity of Glul is weak in the P23H compared to WT. The top and side yellow boxes on each panel show the and X and Y maximum intensity orthogonal projections, respectively. Glul immunostaining is much weaker in the OPL and outer ONL of P23H transgenic compared to the WT (highlighted in white box). (**C**) Relative mRNA level of Glul-a is higher in the WT compared to the P23H (*n* = 3 fish per genotype; error bars are ± SD; * *p* < 0.05).

Glutamine synthetase (Glul) is specifically highly enriched in the Müller glial cells and is involved in neurotransmitter recycling [45]. Examination of Glul immunostaining revealed a prominent change in its expression in P23H compared to the WT. Glul labeling showed decreased intensity in the outer INL and OPL of P23H transgenics, which is seen clearly in orthogonal projections (Figure 8B, highlighted in white box). This is the area most affected by the degenerating rods and proliferating rod progenitor cells, and may reflect changes in glutamate cycling in the retina. This was further confirmed by the significantly reduced Glul-a mRNA level in the transgenic fish compared to the WT (n = 3; *p* = 0.01; Figure 8C). 

## 4. Discussion

In this project, we have developed a transgenic zebrafish model of Retinitis Pigmentosa that displays chronic rod photoreceptor degeneration and continuous regeneration. This model has certain advantages. The mutant protein carries an epitope tag, allowing us to track its expression and localization. As a result, we know that the mutant protein is expressed specifically in the rod photoreceptors, resulting in their degeneration (Figure 2 and Figure 3). The rods in the P23H transgenic have very small, deformed outer segments (Figure 3 and Figure S2), and some of the mutant protein is delocalized over the plasma membrane of the cell. These features closely resemble those observed in many models of RP [15,16,36]. The line has been developed through insertion of a transgene and selective incrossing of individuals whose offspring showed uniformity of expression. Thus, the line contains multiple copies of the transgene. While we have not attempted to investigate the effect of copy number on phenotype, it is expected that a milder phenotype may be obtained by outcrossing with wild type fish and selection for single-copy derivative lines.

Our transgenic model utilizes a 1.8 kb zebrafish rhodopsin promoter and displays initial expression at 3 dpf in cells scattered through the outer retina. This is essentially the same as zebrafish lines expressing either GFP or mCFP from a 5.5 kb *Xenopus* rhodopsin promoter [36,37]. Curiously, at this age, immunostaining with the Retp1 anti-rhodopsin antibody did not detect expression of rhodopsin in the wild type fish (Figure 2), suggesting that the transgenes lack regulatory elements that delay the timing of expression of the native gene. Morris et al. [36] noted that the first-born wave of rods distributed through the retina expressing the mCFP transgene disappear, with subsequent rods being inserted near the retinal margins. This closely matches our observations of the expression pattern of the P23H rhodopsin transgene, and implies a very short lifetime for the rods born in the initial wave. Intriguing also is the appearance of the antigen labeled by the Zpr1 antibody in nascent outer segments of cones at 3 dpf in the P23H transgenic retina, but not in the wild type. While this may simply reflect slight differences in timing of collection of the samples or rate of development in batches of fish, it is also possible that rods expressing the P23H mutant rhodopsin exert a cell non-autonomous effect on surrounding cones. This is a topic that warrants further investigation.

Despite the degeneration of rods, the adult retina continues to harbor some regenerated rods and possibly rods unaffected by the transgene, as well as a largely normal complement of double cones. This is similar to the mCFP transgenic zebrafish model of RP [36]. Furthermore, proliferating progenitor cells are abundant, indicating a high rate of rod regeneration. Even though rods are synthesized continuously, PKC staining of the rod bipolar cells revealed that some contacts were formed with the regenerated rods in the P23H transgenic, along with retention of synapses with cones (Figure 7). We do not see a complete or severe loss of PKC staining as in mammalian models [46,47], which may be because of the continuous regeneration of rods in the zebrafish model. An important question for future research is whether newly-formed rods make functional synapses with these bipolar cells.

Outside of photoreceptors, the Müller glial cells display the most prominent remodeling that we observed in the P23H transgenic retina. The noticed decrease in the levels of glutamine synthetase in the Müller glia might be due to disrupted Müller glial function in the P23H transgenic compared to the WT. Previous studies have shown that loss of major glutamate-releasing neurons in the retina can lead to reduced expression of glutamine synthetase in the Müller cells [45,48]. Important Müller glial cell functions including neurotransmitter recycling, carbon dioxide and potassium siphoning, visual pigment cycling, glycolysis and water regulation could be affected during a continuous degeneration-regeneration scenario [49]. 

A number of retinal degeneration models have previously been established in zebrafish. Forward genetic screens that exploited visual behavior and light response studies in zebrafish led to the identification of novel gene mutations involved in retinal degeneration and also established genetic models to study the pathology [50,51]. The cone-specific phosphodiesterase gene (*pde6c*) mutant was first identified in zebrafish by genetic screening and it leads to the rapid degeneration of all cone photoreceptors soon after their formation [52]. This was followed by the identification of cone degeneration in mice and humans as *pde6c* [53]. Photoreceptor degenerations caused by defects in ciliary transport from the inner to outer segments have been characterized by lines with mutations to *ovl*, *flr*, *ift57*, *ift172*, or *elipsa* genes [54,55,56,57]. Other lines identified by genetic screens utilizing escape response assays were related to night blindness (*nba*, *nbb*, *nbc*, and *nbd* mutant strains) [58,59,60]. These strains, however, were lethal as homozygotes and had a variable degree of degeneration in heterozygous fish, suggesting that the genes had important functions outside of the retina [60]. 

Multiple mutant and transgenic zebrafish lines with photoreceptor degeneration have been produced and characterized. An X-linked RP model has been generated and characterized by mutating the retinitis pigmentosa 2 (RP2) gene. The model revealed that a 12 bp in-frame deletion at the C-terminal end of the protein led to a loss of RP2 protein structural stability [61]. Protein instability was found to be the predominating pathogenic consequence for most RP2 mutations [61,62]. Morris et al. [36] serendipitously developed a transgenic zebrafish XOPS-mCFP line that has selective degeneration of rods and hence can be used as a model to study rod degeneration and regeneration. This model is quite similar to our P23H transgenic model, as has been noted above. The two models have slightly different attributes, making each useful in certain paradigms for studies of photoreceptor degeneration and regeneration in the context of RP. 

The greatest utility of the P23H transgenic zebrafish model of RP will be in studies of regeneration of photoreceptors. Several previous studies have used acute damage models to characterize the regenerative properties of zebrafish retina. Many studies have inflicted retinal damage via pharmacological toxins, light-induced injury, or physical excision [41,63,64]. These studies are particularly useful to identify cell types and mechanisms involved in regeneration. Acute damage studies are limited by the time of recovery and regeneration; depending on the type and extent of insult, recovery may take weeks. However, this may not accurately represent the regenerative process in chronic degenerative diseases such as RP. Our results clearly show that this P23H rhodopsin model of RP exhibits chronic rod degeneration as well as regeneration, which makes it a useful model to study the regeneration of retinal neurons during RP. 

Regeneration of both rod and cone photoreceptors in zebrafish can occur from differentiated Müller glial cells that enter the cell cycle and produce retinal progenitors that can differentiate as either rods or cones [65]. Indeed, in acute light- or chemical-induced damage models, many proliferating cells are seen in the INL [41,63], revealing the Müller cell origin of the regenerative response. In contrast, most of the proliferating cells in our P23H rhodopsin model are seen in the ONL, with very few cells in the INL. There may be two possibilities to account for this difference: (i) a small number of cells in the INL give rise to many rod progenitors, or (ii) there may be resident rod progenitors always remaining in the retina that proliferate during rod photoreceptor degeneration, avoiding the requirement for Müller glia dedifferentiation followed by proliferation [65,66]. A comparison of chronic rod degeneration in the mCFP transgenic zebrafish and chronic cone degeneration in the *pde6c^W59^* mutant zebrafish found these mechanisms to be different, with cone degeneration stimulating proliferation of Müller cell-derived stem cells while rod degeneration promoted proliferation of a dedicated rod progenitor cell in the ONL [66]. Although we have not examined the mechanisms of regeneration in the P23H transgenic model in detail, our observations are congruent with this interpretation of chronic rod regeneration. Overall, the P23H rhodopsin transgenic zebrafish provides an elegant system to study not only the degeneration mechanisms but also the regeneration mechanisms in an RP model.

## Figures and Tables

**Table 1:** List of antibodies used in this study.

Antibody	Host	Antigen	Source	Catalog Number	Dilution
Retp1	Ms	Rat Rhodopsin	Novus Biologicals	NB120-3267-0	1:200
			Centennial, CO, USA		
Flag-DDK	Ms	DYKDDDDK	Origene	TA50011	1:250
			Rockville, MD, USA		
Zpr1/Fret43	Ms	Fixed Zebrafish retinal cells	ZIRC	AB_10013803	1:10
			Eugene, OR, USA		
PCNA	Ms	Protein A-Proliferating Cell Nuclear Antigen fusion protein	Abcam Cambridge, MA, USA	ab29	1:100
PCNA	Rb	Synthetic peptide corresponding to Human PCNA aa 200 to the C-terminus	Abcam	Ab18197	1:100
SV2	Ms	Synaptic Vesicle Protein 2a	Developmental Studies Hybridoma Bank	SV2	1:100
			Iowa City, IA, USA		
GS-6	Ms	Glutamine Synthetase	Millipore-Sigma	MAB302	1:1000
			Burlington, MA, USA		
BU-1	Ms	5-bromo-2-deoxyuridine (BrdU)	Invitrogen	MA3-071	1:100
			Carlsbad, CA, USA		
PKC-α	Rb	Protein Kinase Cα	Millipore, Sigma	P4334	1:400
Cy3	Gt	Goat Anti-Mouse IgG Fcγ subclass 2a specific	Jackson ImmunoResearch	115-165-206	1:500
			West Grove, PA, USA		
Alexa Flour 488	Dk	Donkey Anti-Mouse IgG (H+L)	Jackson ImmunoResearch	715-545-150	1:500
Alexa Fluor 488	Dk	Donkey Anti-Rabbit IgG	Jackson ImmunoResearch	711-545-152	1:500
Alexa Fluor 488	Gt	Goat Anti Mouse IgG Fcγ subclass 1 specific	Jackson ImmunoResearch	115-545-205	1:500
DAPI		Nuclear Counterstaining	Vector Laboratories	H-1000	
			Burlingame, CA, USA		

**Table 2:** Primers used for real-time PCR.

Gene	Primer Sequence (5′-3′)	Size (bp)	GenBank Accession
*prkca*-FP	TCCCCAGTATGTGGCTGGTA	119	NM_001256241.1
*prkca*-RP	TTGGCTATCTCAAATTTCTGTCG		
*glula*-FP	CGCATTACAGAGCCTGCCTA	212	NM_181559.2
*glula*-RP	ATTCCAGTTGCCTGGGATCG		
*GAPDH*-FP	ATGACCCCTCCAGCATGA	134	NM_213094.2
*GAPDH*-RP	GGCGGTGTAGGCATGAAC		

## Supplementary Materials

The following are available online at https://www.mdpi.com/2073-4409/9/10/2242/s1, Figure S1: Genotyping PCR for P23H transgenic, Figure S2: Retp1 antibody cross-reactivity to double cones, Figure S3: TUNEL labeling in INL and RGC layer, Figure S4: PKC labeling is weak in P23H mutant rod bipolar cells.
