# Supplementary material for: A Zebrafish Model of Retinitis Pigmentosa Shows Continuous Degeneration and Regeneration of Rod Photoreceptors
Source: Cells. 2020 Oct 6;9(10):2242. doi: 10.3390/cells9102242 (PMC7599532; doi:10.3390/cells9102242)
Supplement: Supplementary file 1 [file cells-09-02242-s001.pdf]

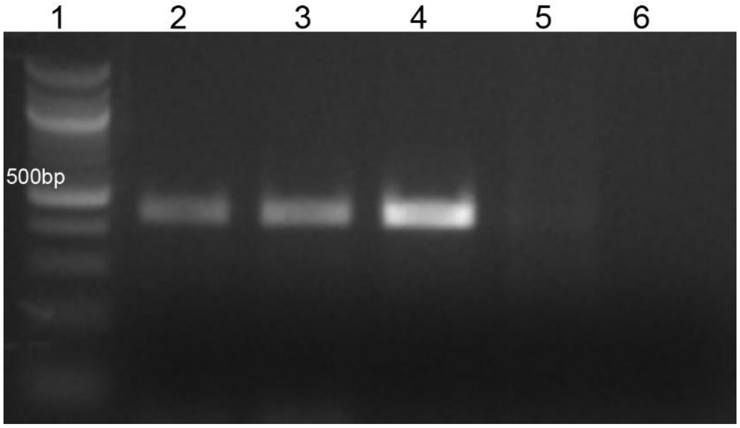


Figure S1. Genotyping PCR for P23H mutant rhodopsin. Lane 1 - 100 base pair ladder; Lanes 2,3,4 - P23H mutant rhodopsin specific PCR product (size 447 bp), Lane 5 - WT, Lane 6 - Negative control (No template).

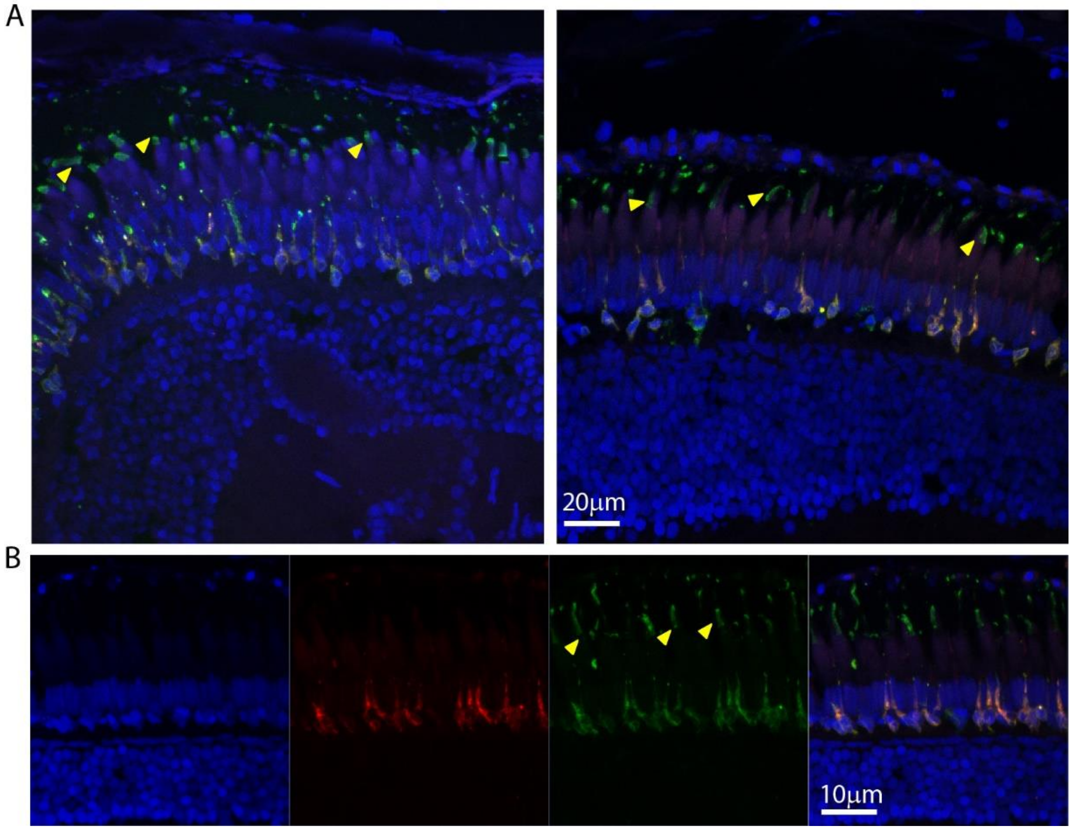


Figure S2. Rhodopsin antibody Retp1 binds to double cone opsins as well as rhodopsin. (A) Retp1 immunostaining (green) in adult P23H transgenic zebrafish retina localized to one double cone outer segment (yellow arrowheads) associated with each double cone myoid, labeled with DAPI. Rhodopsin labeling in rods is apparent as co-localization with Flag-tagged P23H rhodopsin (red). (B) Another set of images with individual panels show the localization of the Retp1 signal at the outer segment of the double cones (yellow arrowheads). Retp1-green, Flag-red, DAPI-blue.

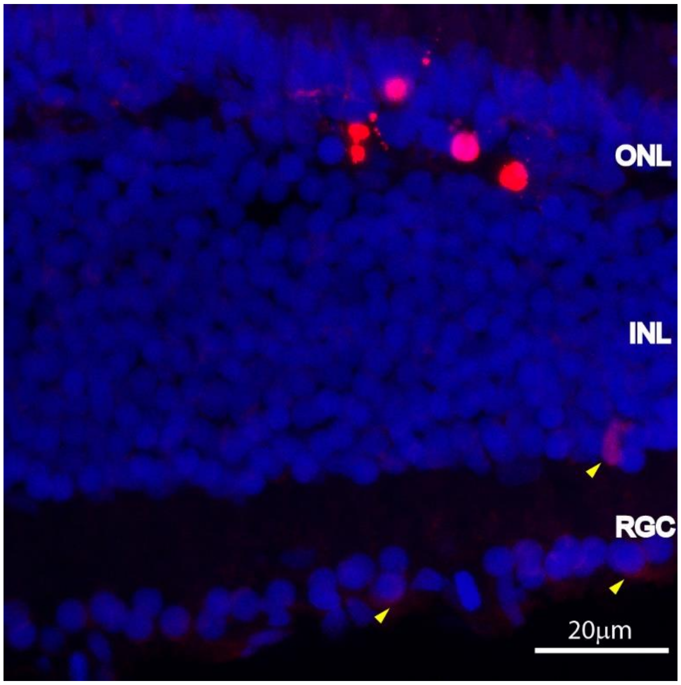


Figure S3. TUNEL-positive cells (red) are occasionally seen in the inner nuclear layer (INL) and retinal ganglion cell layer (RGC; yellow arrowheads) in the P23H transgenic zebrafish retina.

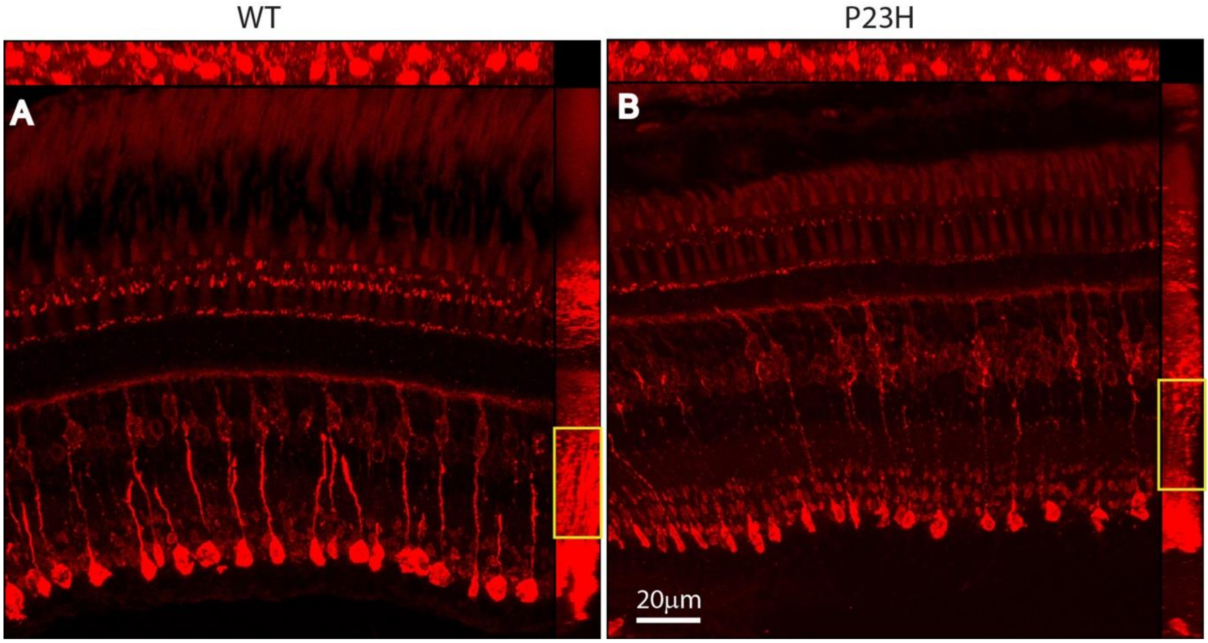


Figure S4. PKC labeling is weak in P23H transgenic rod bipolar cells, and weakly present in cone On bipolar cells. Orthogonal projection of the maximum intensity projection of PKC immunostaining show a marked decrease of PKC staining in the P23H transgenic (B) compared to wild type (A). The yellow box highlights the region of notable difference in the y-axis of the orthogonal projection.
